# College and Hospital Notes

**Published:** 1902-01

**Authors:** 


### COLLEGE AND HOSPITAL NOTES.

McGill University is to have a foundation on political economy, to be known as the William McKinley fellowship, for which the American residents of Montreal have subscribed a fund of $9,000. This is the most befitting kind of a monument to build to the memory of the martyr President. Dr. Thomas G. Roddick, has been appointed dean of the medical faculty of McGill, replacing Dr. Craik, resigned, on account of age.

---

The Buffalo Hospital of the Sisters of Charity has received from Dr. Edward J. Meyer, of Buffalo, a present of an operating table and instrument cabinet. The table was manufactured by the Kny-Scherer Company, of New York, and was on exhibition in the Manufacturers' Building at the Pan-American Exposition. It is made of glass and steel and is valued at $250. The instrument cabinet is also made of glass and steel and is the one that was used at the Emergency Hospital on the Exposition grounds.

The Sisters of Charity have purchased the three sterilising machines that were in the exhibit at the Pan-American. They are made of nickel and copper and are as perfect as can be made.

---

The University of the State of New York in its authorised announcements for December, 1901, gives some interesting data, from which we extract the following:

College Department.—Report of the college department for the year ending July 31, 1901, will show that during the nine years in which the medical licensing examinations have been held 6,349 physicians have been examined, of which number 78 per cent. were licensed. At the dental licensing examinations, which have been given for 6 years, 1,264 dentists have been examined, of which number 79 per cent. were licensed. At the veterinary licensing examinations, which have also been held for 6 years, 93 veterinarians have been examined, of which number 56 were licensed. During the 5 years in which examinations for expert public accountants have been held, 79 have been examined, of which number 58 received certificates. In these statistics each candidate who fails is counted as often as examined.

During the last year, in medicine, 671 old school candidates, 58 homeopaths and 20 eclectics have been examined. There were no failures in the case of graduates of 6 of the New York medical schools, and in only 2 of the New York schools did the percentage of accepted candidates fall below 90. In 1901, of the 194 examined in dentistry, 161 received licenses; of the 12 examined in veterinary medicine 7 received licenses; of the 17 examined in public accounting 13 received certificates as certified public accountants.

Award at Pan-American Exposition.—The university was awarded a gold medal for exhibit of educational methods and results. A diploma and a bronze form of the medal is to accompany award.

---

THE State Hospital for Consumptives has finally been located at Raybrook, between Saranac and Lake Placid. Thus a long controversy comes to an end, and if the site chosen has not the advantages of the one first selected by the commission, it is an eligible one and should prove satisfactory. Dr. John H. Pryor, the commissioner from Buffalo, who was the chief mover in the establishment of the hospital, has expressed himself as satisfied with Raybrook.

---

A MARINE hospital in Buffalo will be an accomplished fact in the near future. At least it will be if the supervising surgeon-general of the service and the Hon. D. S. Alexander, M. C. from Buffalo, can bring it about. The Secretary of the Treasury has approved the recommendations of the surgeon-general and Representative Alexander has introduced a bill in Congress appropriating $125,000 for the hospital.

The subject has been agitated for some time. At present sailors are treated in one of the local hospitals, where there is a so-called marine ward. The importance of Buffalo as a lake port demands that a special government hospital be built here. These hospitals are largely supported by the sailors themselves, who contribute a certain percentage of their monthly pay for the purpose. In return they receive free treatment.

